# Synergistic Therapeutic Potential of Dual 3D Mesenchymal Stem Cell Therapy in an Ischemic Hind Limb Mouse Model

**DOI:** 10.3390/ijms241914620

**Published:** 2023-09-27

**Authors:** Dong-Sik Chae, Sang Joon An, Seongho Han, Sung-Whan Kim

**Affiliations:** 1 Department of Orthopedic Surgery, College of Medicine, Catholic Kwandong University, International St. Mary’s Hospital, Incheon 22711, Republic of Korea; 2 Department of Neurology, College of Medicine, Catholic Kwandong University, International St. Mary’s Hospital, Incheon 22711, Republic of Korea; 3 Department of Family Medicine, College of Medicine, Dong-A University, Busan 49236, Republic of Korea; 4 Department Medicine, College of Medicine, Catholic Kwandong University, Gangneung 25601, Republic of Korea

**Keywords:** adipose mesenchymal stem cells, angiogenic potential, 3D culture systems, regenerative medicine, tissue engineering

## Abstract

Three-dimensional (3D) culture systems have been widely used to promote the viability and metabolic activity of mesenchymal stem cells (MSCs). The aim of this study was to explore the synergistic benefits of using dual 3D MSC culture systems to promote vascular regeneration and enhance therapeutic potential. We used various experimental assays, including dual 3D cultures of human adipose MSCs (hASCs), quantitative reverse transcription polymerase chain reaction (qRT-PCR), in vitro cell migration, Matrigel tube network formation, Matrigel plug assay, therapeutic assays using an ischemic hind limb mouse model, and immunohistochemical analysis. Our qRT-PCR results revealed that fibroblast growth factor 2 (FGF-2), granulocyte chemotactic protein-2 (GCP-2), and vascular endothelial growth factor-A (VEGF-A) were highly upregulated in conventional 3D-cultured hASCs (ASC-3D) than in two-dimensional (2D)-cultured hASCs. Hepatocyte growth factor (HGF), insulin-like growth factor-1 (IGF-1), and stromal-cell-derived factor-1 (SDF-1) showed higher expression levels in cytokine-cocktail-based, 3D-cultured hASCs (ASC-3Dc). A conditioned medium (CM) mixture of dual 3D ASCs (D-3D; ASC-3D + ASC-3Dc) resulted in higher migration and Matrigel tube formation than the CM of single 3D ASCs (S-3D; ASC-3D). Matrigel plugs containing D-3D contained more red blood cells than those containing S-3D. D-3D transplantation into ischemic mouse hind limbs prevented limb loss and augmented blood perfusion when compared to S-3D transplantation. Transplanted D-3D also revealed a high capillary density and angiogenic cytokine levels and transdifferentiated into endothelial-like cells in the hind limb muscle. These findings highlight the benefits of using the dual 3D culture system to optimize stem-cell-based therapeutic strategies, thereby advancing the therapeutic strategy for ischemic vascular disease and tissue regeneration.

## 1. Introduction

Vascular ischemic diseases stem from the stenosis or obstruction of arterioles and capillaries and lead to a diminished blood supply and subsequent deprivation of oxygen and vital nutrients necessary for cellular metabolism. The primary cause of ischemia is the blockage of arterial blood flow to a tissue, organ, or extremity. Ischemia encompasses a range of conditions resulting from ischemic injury, such as ischemic heart or vascular disease and ischemic cerebral disease [1], which present a significant global public health concern owing to the high rates of mortality and disability [2] and are the leading causes of disability and death.

For decades, traditional cytokine-based therapies have been used in medical research and clinical studies to induce therapeutic angiogenesis [3]. However, administered recombinant human vascular endothelial growth factor (VEGF) in patients with stable exertional angina did not yield significant differences compared to the administered placebo for improving myocardial perfusion [4]. In recent years, cell-based therapies have attracted attention as promising methods for treating ischemic diseases. Stem cells are capable of differentiating into specialized cell types and are derived from various tissues, such as adipose tissue, cord blood, bone marrow, and placental tissue. Stem cell therapy can potentially promote angiogenesis and effectively treats ischemic diseases [5]. However, it has certain limitations, including limited therapeutic efficacy [6,7].

Three-dimensional (3D) spheroid cultures prevent apoptosis and promote cell stabilization [8]. The improved cell behavior observed in 3D cultures can be attributed to several factors. First, the 3D environment provides spatial organization and cell–cell interactions that resemble the in vivo tissue architecture. This structural arrangement allows for more optimized nutrient and oxygen diffusion throughout the cell population, thereby promoting cell viability and metabolic activity. Three-dimensional-cultured spheroids from mesenchymal stem cells (MSCs) enhance paracrine factors, thus exhibiting improved therapeutic efficacy [9,10,11]. Preconditioning human adipose-derived mesenchymal stem cells (hASCs) in 3D aggregates in a hypoxic environment results in the upregulation of hypoxia-inducible factor-1α and enhanced secretion of angiogenic factors, such as fibroblast growth factor (FGF)-2, VEGF, and hepatocyte growth factor (HGF) [12,13]. These effects are more pronounced than those of traditional monolayer two-dimensional (2D) culture systems [12].

In our previous study, we demonstrated that novel hASC culture conditions containing a combination of potent growth factors enhanced the antifibrotic potential of the liver [14]. This cytokine combination stimulated hASCs, leading to a robust angiogenic capacity. Recently, we developed a novel and straightforward 3D culture system to enhance the angiogenic potential [15]. By harnessing the regenerative properties of conventional stem cell therapy, we aimed to investigate the synergistic therapeutic effects of the co-transplantation of 3D hASCs and elucidate the mechanisms underlying an ischemic mouse hind limb model.

## 2. Results

### 2.1. Cell Morphology and Angiogenic Property of 2D or Dual 3D Adipose-Derived Mesenchymal Stem Cells (ASCs)

**Figure 1:**
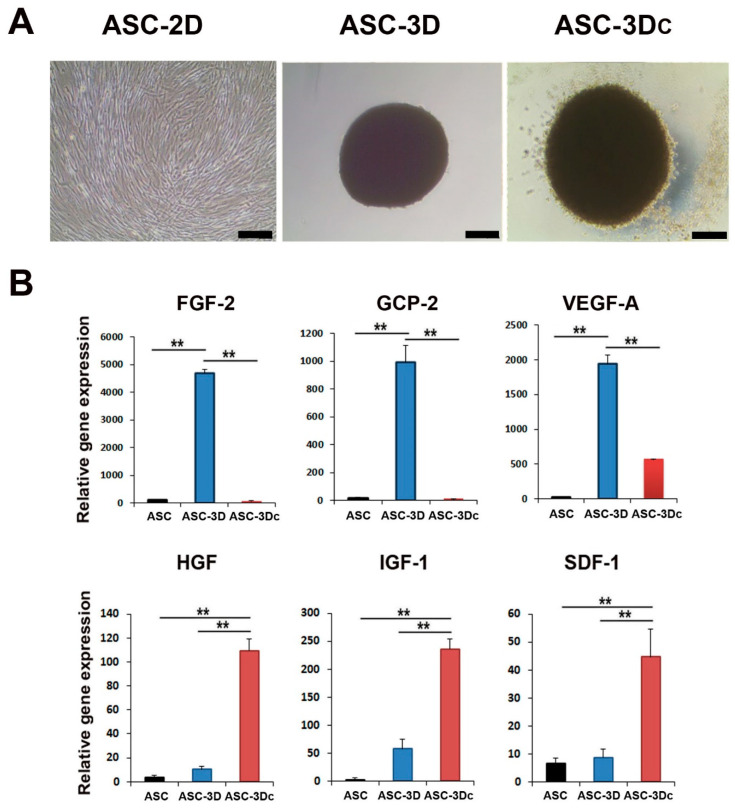
Analysis of morphology and proangiogenic gene expression in two-dimensional (2D) or three-dimensional (3D) adipose-derived mesenchymal stem cells (ASC). (**A**) Morphology of 2D ASCs, ASC-3D, and 3D-cultured hASCs (ASC-3Dc). Bars = 100 μm. (**B**) Quantitative reverse transcription polymerase chain reaction (qRT-PCR) was used to measure gene expression patterns of multiple factors. Individual values were normalized to the expression level of glyceraldehyde-3-phosphate dehydrogenase (GAPDH). ** *p* < 0.01; n = 4 per group.

The morphologies of spindle-like 2D-cultured, adipose-derived mesenchymal stem cells (ASC-2D) and 3D-spheroid-cultured ASCs without cytokines (ASC-3D) or with a cytokine cocktail (ASC-3Dc) containing epidermal growth factor (EGF), FGF-2, insulin-like growth factor-1 (IGF-1), and VEGF-A are shown in Figure 1A. ASC-3Dc grew larger than ASC-3D, indicating that the cytokine cocktail induced cell proliferation.

To evaluate the angiogenic properties of the two types of 3D-cultured ASCs, the expression levels of proangiogenic genes were examined using qRT-PCR. Dual 3D-cultured ASCs were compared with ASC-2D control cells. The expression levels of FGF-2, granulocyte chemotactic protein-2 (GCP-2), and VEGF-A were higher in ASC-3D mice than in ASC-2D mice. However, IGF-1, stromal cell-derived factor-1 (SDF-1), and HGF were highly expressed in ASC-3Dc compared to ASC-3D and ASC-2D (Figure 1B). Therefore, we hypothesized that the combination of these two types of 3D ASCs might influence their synergistic therapeutic potential in an ischemic hind limb model.

### 2.2. Secretions from Dual ASC-3D and ASC-3Dc (D-3D) Enhance Cell Migration and Tubular Network Formation

**Figure 2:**
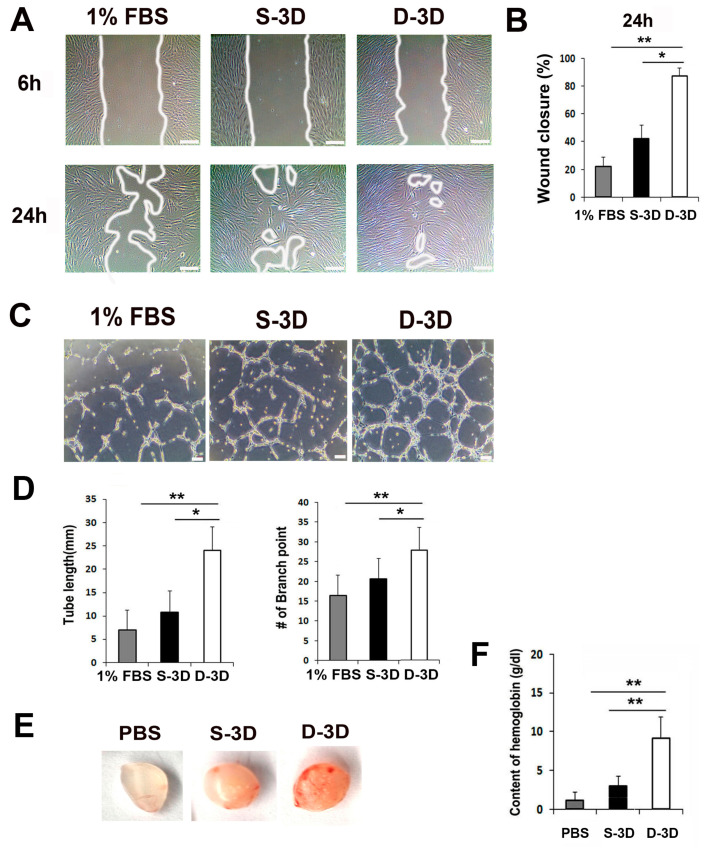
In vitro and in vivo angiogenic properties of dual 3D ASCs (D-3D). (**A**) Representative photograph of scratch wound migration. Bars = 100 μm. (**B**) The culture medium (CM) of D-3D highly improved wound closure through human dermal fibroblasts (HDFs) when compared to the CM of single 3D ASCs (S-3D). * *p* < 0.05, ** *p* < 0.01; n = 5 per group. (**C**) Representative photograph of Matrigel tube formation. Bars = 100 μm. (**D**) Quantification of branching point and tube length. The CM of D-3D significantly increased the formation of a tubular structure when compared to the CM of S-3D. ** *p* < 0.01; n = 5 per group. (**E**) Representative photograph of Matrigel plugs injected with S-3D and D-3D at 14 days after cell injection. (**F**) Quantification of hemoglobin content. D-3D injection significantly increased the hemoglobin content when compared to S-3D. ** *p* < 0.01; n = 4 per group.

To examine whether factors secreted from the mixed culture medium (CM) of dual ASC-3D and ASC-3Dc (D-3D) promote cell migration, tube formation, scratch wound cell migration, and Matrigel tube formation assays were performed. First, ASC-3D and ASC-3Dc spheroids were generated for 5 days using a cytokine cocktail, and each cell line was cultivated in low-glucose DMEM containing 1% FBS using a suspension cell culture system for 3 days. The CM of each cell type was collected and used for the experiments. ASC-3D is a well-established and conventional 3D culture system. Therefore, we used this 3D system as our S-3D control group. D-3D consist of a 1:1 ratio of CM of ASC-3D and ASC-3Dc. The scratch wound closure assay revealed that the CM of D-3D significantly accelerated the rate of fibroblast migration compared to that of S-3D (Figure 2A,B). In addition, mixed CM of dual ASC-3D and ASC-3Dc (D-3D) induced significantly higher tube lengths and branching points than the CM of single ASC-3D (S-3D) (Figure 2C,D). These results indicate that the CM of D-3D provides a favorable environment for the angiogenic activity of host cells.

To evaluate the in vivo angiogenic potential of D-3D, a Matrigel plug assay was performed. The D-3D Matrigel plug was generated by mixing ASC-3D and ASC-3Dc (each 5 × 10^5^ cells in 500 μL of Matrigel), and the S-3D Matrigel plug was generated by mixing 1 × 10^6^ cells in 500 μL of Matrigel. We also used a PBS control group by combining 50 μL of PBS with 500 μL of Matrigel. Next, the Matrigel plugs were subcutaneously transplanted into nude mice; they were collected after 2 weeks, and hemoglobin levels were examined. Interestingly, significantly higher numbers of red blood cells were detected in the D-3D Matrigel plugs than in the S-3D plugs (Figure 2E,F). No red blood cells were detected in the control group. These data suggest that the secreted factors derived from D-3D have high angiogenic potential.

### 2.3. D-3D Shows Robust Therapeutic Potential in Hind Limb Ischemia (HLI)

**Figure 3:**
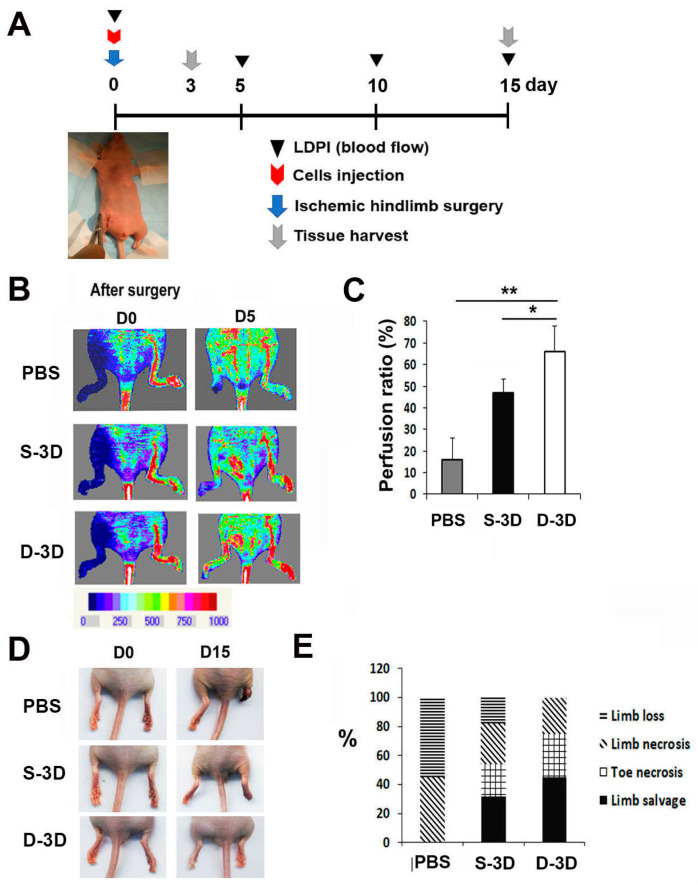
Analysis of the angiogenic therapeutic effects of D-3D in an ischemic hind limb model. (**A**) A schematic of the experimental protocol for ischemic hind limb surgery, cell transplantation, LDPI analysis, and subsequent tissue collection. (**B**) Representative LDPI images depicting the measurement of blood flow recovery in ischemic hind limbs following cell injection. (**C**) Quantitative analysis of blood perfusion conducted 2 weeks after cell injection. n = 7 per group. ** *p* < 0.01; * *p* < 0.05. (**D**) Representative images of limb salvage, limb necrosis, or limb loss after cell transplantation. (**E**) Quantitative analysis of limb salvage, limb necrosis, or limb loss after cell injection.

To investigate the therapeutic potential of D-3D in ischemic disease, HLI was induced in nude mice [16]. For this, D-3D (mixture of ASC-3D and ASC-3Dc, each 5 × 10^5^ cells), S-3D (ASC-3D, 1 × 10^6^ cells), or PBS was injected intramuscularly into the ischemic hind limb area after ligation of the femoral artery (Figure 3A). Laser Doppler perfusion imaging revealed that the recovery rate of blood perfusion in D-3D-injected limbs was significantly higher than that in limbs injected with S-3D or PBS on day 5 (Figure 3B,C). In addition, D-3D-injected mice exhibited less limb loss and a higher limb salvage ratio than the S-3D or PBS control groups 4 weeks after cell injection (Figure 3D,E).

### 2.4. D-3D Injection Enhances Capillary Density in a Hind Limb Mouse Model

**Figure 4:**
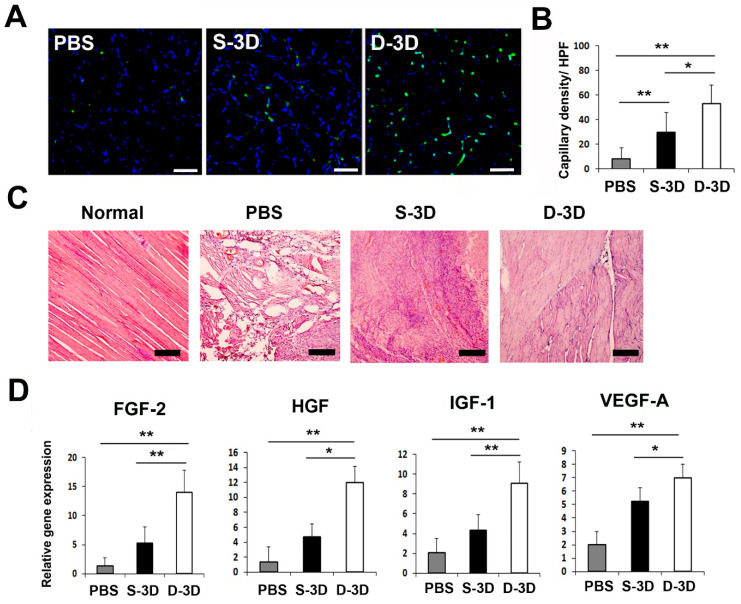
Analysis of the therapeutic mechanism. (**A**) Representative images showing the capillary density in hind limb tissues at 4 weeks after cell injection. The blue stain represents nuclear 4′,6-diamidino-2-phenylindole (DAPI), while the green stain represents interleukin beta 4 (ILB4). Bars = 500 μm. (**B**) Quantitative analysis of the capillary density in hind limb tissues after cell injection. Statistical analysis showed significant differences between groups, indicated as ** *p* < 0.01 and * *p* < 0.05. n = 6 per group. (**C**) Histological images of hind limb tissues stained with hemolysin and eosin (H&E) at 15 days after cell injection. Bars = 200 μm. (**D**) Analysis of angiogenic factor expression in hind limb tissues 3 days after cell transplantation. n = 7 per group. ** *p* < 0.01; * *p* < 0.05.

To elucidate the therapeutic mechanism that contributes to the improved recovery of blood flow and the reduced rate of lower limb loss or the increased rate of limb salvage after D-3D transplantation, we analyzed the capillary density through histological staining using the endothelial cell marker interleukin beta 4 (ILB4). The capillary density was significantly higher in D-3D-injected mice than in S-3D-injected mice and PBS-treated control mice on day 7 (Figure 4A,B). Hemolysin and eosin (H&E) staining revealed that D-3D transplantation resulted in reduced muscle degeneration and lower infiltration of inflammatory cells compared to the PBS-treated control and S-3D treated mice (Figure 4C).

To investigate the therapeutic mechanisms that contribute to the increased rate of limb salvage, the expression levels of angiogenic factors in hind limb tissues after cell injection were measured using qRT-PCR. The results revealed that D-3D-injected limbs showed significantly upregulated FGF-2, HGF, IGF-1, and VEGF-A compared to S-3D- or PBS-treated hind limb tissues 5 days after cell injection. These data indicate that D-3D injection promotes multiple angiogenic biological factors for vascular protection or regeneration in vivo (Figure 4D).

### 2.5. 3D ASCs Differentiated into Endothelial Cells In Vivo

**Figure 5:**
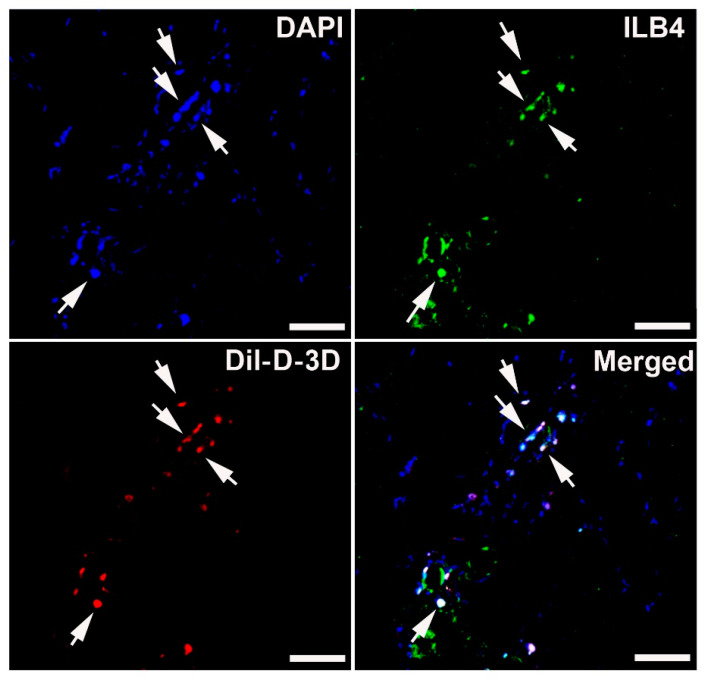
Engraftment and endothelial differentiation property of D-3D in an ischemic hind limb model. Representative images of co-localized Dil-labeled D-3D in the ischemic hind limb 4 weeks after cell injection. Nuclei were stained with DAPI (blue). ILB4 (green), Dil (red). Arrows indicate ILB4 and Dil double-positive cells, suggesting endothelial differentiation of D-3D. Bars = 500 μm.

To investigate the endothelial differentiation potential of 3D ASCs, we conducted an in vivo study using a mouse model of induced HLI. Specifically, 1 × 10^6^ Dil-labeled D-3D were directly injected into the hind limb adductor muscle, which is an ischemic area. After 4 weeks, hind limb muscle tissues were collected for analysis. Immunohistochemistry revealed the co-localization of injected D-3D with ILB4, an endothelium-specific marker, in the treated limbs. These data suggest that injected 3D-ASCs can differentiate into endothelial cells in vivo (Figure 5).

## 3. Discussion

Peripheral artery disease (PAD) and other ischemic disorders pose significant challenges in terms of treatment and tissue regeneration. The combination of different stem cell populations in a 3D environment likely enhances paracrine signaling, trophic support, and angiogenic potential, leading to improved tissue regeneration and functional outcomes. In this study, we investigated the synergistic effects of dual 3D stem cell therapy and the mechanisms underlying the observed therapeutic effects. To the best of our knowledge, this is the first study that demonstrates the synergistic angiogenic properties of dual 3D stem cell therapy.

Paracrine activity plays a crucial role in the therapeutic mechanisms of stem cells in tissue repair and regeneration [17]. However, traditional 2D culture conditions limit the paracrine activity of stem cells, ultimately affecting their therapeutic efficacy. This can lead to suboptimal secretion of essential growth factors, cytokines, and other paracrine signaling molecules. Moreover, 2D culture conditions may not accurately mimic the complex and dynamic interactions between cells and their surrounding extracellular matrix, which are essential for maintaining the regenerative properties of stem cells. These limitations can hamper the ability of stem cells to exert their full therapeutic potential when transplanted in vivo.

Recently, improved cell behavior was observed in 3D culture systems [18]. This could be attributed to several factors. First, the 3D environment offers spatial organization and cell–cell interactions that closely resemble in vivo tissue architecture [19]. This structural arrangement allows for more optimized nutrient and oxygen diffusion throughout the cell population, thereby promoting cell viability and metabolic activity. Second, 3D culture systems often use biomaterial scaffolds or hydrogels that mimic the extracellular matrix (ECM) composition and provide mechanical support [20]. These scaffolds can be functionalized with bioactive molecules or growth factors to create a microenvironment that stimulates cell proliferation and survival. Additionally, the signaling pathways involved in cell adhesion, migration, and proliferation are regulated differently in 3D than in 2D cultures. Altered mechanical and biochemical cues in 3D environments can activate specific signaling pathways, such as integrin-mediated signaling and mechanotransduction pathways, which contribute to enhanced cell viability and proliferation [21]. Three-dimensional culture systems enhance the paracrine activity of stem cells by producing angiogenic, anti-apoptotic, and anti-inflammatory factors [15,22,23]. These 3D culture systems have demonstrated favorable therapeutic effects in various diseases, such as liver fibrosis, myocardial infarction, kidney injury, and peritonitis [12,24,25,26]. In our study, qRT-PCR data revealed the distinct angiogenic properties of the two different types of 3D-cultured ASCs. Building on this finding, our objective was to present compelling evidence of the synergistic therapeutic potential of dual 3D stem cell therapy in an ischemic hind limb model.

The therapeutic improvement of ischemic cardiovascular disease through stem cell therapy is limited by several factors, including poor engraftment, low angiogenic capacity, and a minimal survival rate in the ischemic environment [27]. These limitations significantly affect the overall efficacy of stem cell therapy. The major therapeutic effects of stem cells occur via paracrine mechanisms [17]. Given the importance of cell migration and tube formation in angiogenesis [28], these properties were evaluated in this study. Similar to results of qRT-PCR data, the CM of D-3D was found to significantly enhance fibroblast migration. In in vitro vasculogenesis assays using Matrigel, treatment with D-3D CM resulted in the robust formation of a well-developed tube network. Moreover, Matrigel plugs containing D-3D in mice exhibited a high red blood cell content, suggesting enhanced vasculogenic capacity. High VEGF-A expression levels in D-3D may contribute to the formation of microvessel-like networks by interacting with another paracrine factor, HGF [29]. Additionally, the elevated expression of SDF-1 derived from D-3D may promote angiogenesis or synergize with GCP-2 to enhance angiogenic processes.

Consistent with the in vitro findings, the in vivo results from treatment injection in the ischemic hind limb model demonstrated favorable therapeutic effects of D-3D transplantation. Notably, D-3D transplantation facilitated limb salvage and accelerated blood perfusion when compared to S-3D transplantation in the ischemic hind limb. This suggests its potential as an effective treatment for ischemic vascular conditions. Histological analysis further supported these positive outcomes, revealing that D-3D-injected limb tissue exhibited lower muscle degradation and higher capillary density than the control group. qRT-PCR results in tissues showed that D-3D-injected limb tissue highly expressed FGF-2, HGF, IGF-1, and VEGF-A. VEGF-A and FGF-2 are well-known pro-angiogenic factors that facilitate the growth of new blood vessels [30]. IGF-1 and HGF also play a crucial role in promoting cell growth, proliferation, and tissue repair [31,32]. These factors might stimulate angiogenesis, enhance the survival of endothelial cells, and contribute to the improvement of ischemic conditions by promoting angiogenesis, tissue regeneration, and cell survival. In therapeutic applications, combinations of these factors or their synergistic effects may maximize their benefits in treating ischemia-related disease. These findings suggest that D-3D transplantation promotes tissue preservation and neovascularization, which are crucial for tissue repair and regeneration under ischemic conditions.

**Figure 6:**
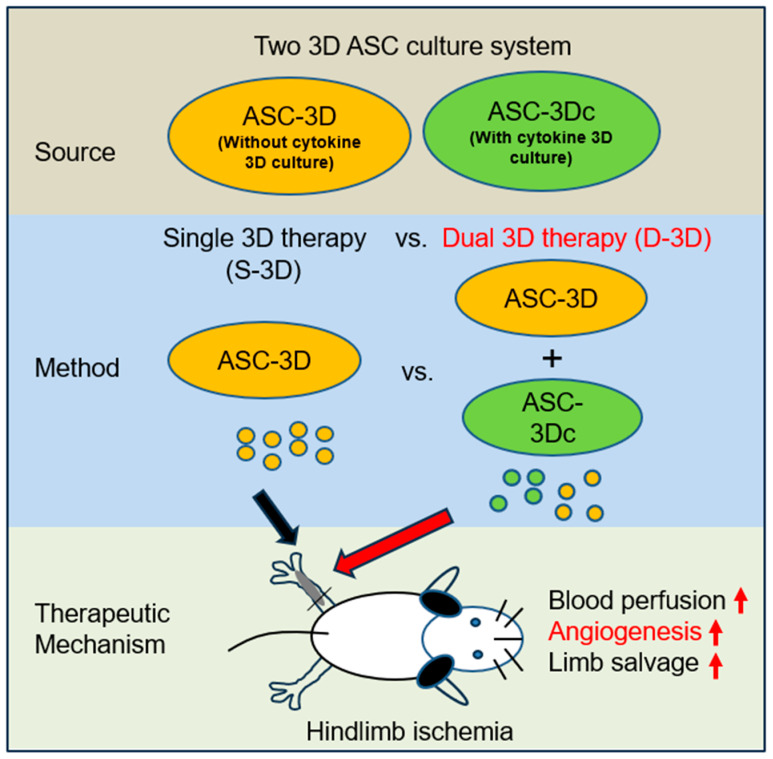
D-3D-based therapy strategy to promote angiogenesis. D-3D therapy exhibited superior angiogenic activity in vitro when compared to S-3D therapy. In a mouse HLI model, D-3D therapy resulted in more substantial limb ischemia repair compared to S-3D therapy. Mechanistically, the injected D-3D therapy induced higher neovascularization. Arrows indicating an increase.

Previous studies have also demonstrated the potent synergistic effects of angiogenic factors, including VEGF-A, FGF-2, and platelet-derived growth factor-b (PDGF-b), on promoting neovascularization [33,34]. Additionally, dual 3D stem cell therapy approaches, such as those using early/late endothelial progenitor cells, ASCs, stromal vascular fraction (SVF), and induced pluripotent stem-cell-derived cardiomyocytes, have shown synergistic improvements in cardiac function and vascular regeneration under ischemic conditions [35,36,37]. Our findings align with those of previous studies, thus confirming the significant advantages of dual 3D stem cell therapy in achieving high therapeutic effects. In this study, we demonstrated that the combination of two types of stem cells cultured in a 3D environment yields superior therapeutic outcomes when compared to single 3D stem cell therapy (Figure 6).

Another therapeutic mechanism of stem cells is their potential for transdifferentiation. Recent studies have demonstrated enhanced endothelial cell differentiation in vitro [23] and vascular regeneration of 3D-cultured ASCs in vivo [38]. In line with these reports, we observed engrafted D-3D expressing an endothelial marker (ILB4) in the mouse hind limb, suggesting endothelial transdifferentiation. These findings suggest that ASC-3Dc possess a high transdifferentiation capacity, which may contribute to the regeneration of damaged tissue and the recovery of function.

In conclusion, our study demonstrated the enhanced angiogenic potential achieved by the application of dual 3D-cultured stem cells. This is a novel and simple idea for enhancing the angiogenic potential of stem cells without genetic modification. The synergistic effects observed in our investigation suggest that dual 3D stem cell therapy is a potential therapeutic approach for vascular tissue regeneration. Nevertheless, to ascertain the clinical efficacy of dual 3D-cultured ASCs in ameliorating ischemic vascular diseases, further investigations and clinical trials are required. These findings will help establish the safety and effectiveness of this therapeutic strategy for future clinical applications.

## 4. Materials and Methods

### 4.1. 2D Culture

Three strains of human ASCs, human vascular endothelial cells (HUVECs), and human dermal fibroblasts (HDFs) were purchased from Thermo Scientific Inc. (Rockford, IL, USA) and maintained at 37 °C under 5% CO_2_ in culture medium (α-minimum essential medium (α-MEM), 10% fetal bovine serum (FBS), 100 U/mL of penicillin, and 100 mg/mL of streptomycin).

### 4.2. Culture with a Cytokine Cocktail

ASCs were cultured in a medium composed of α-MEM supplemented with 2% FBS, 100 U/mL of penicillin, and 100 mg/mL of streptomycin. The culture medium was further supplemented with a cytokine cocktail consisting of 20 ng/mL of human vascular endothelial growth factor (VEGF)-A, 100 ng/mL of human fibroblast growth factor (FGF)-2, 20 ng/mL of human epidermal growth factor (EGF), and 20 ng/mL of human insulin-like growth factor (IGF)-1 for 5 days.

### 4.3. 3D Culture

To generate 3D spheroids, ASCs (5 × 10^4^) were suspended in 27 µL of 10% FBS-containing conventional culture medium or the cytokine cocktail culture medium using the method described by Bartosh et al. [26]. The cell suspension was placed on the cover of culture plates and cultured in an inverted manner for 5 days. To dissociate the cells, the spheroids were incubated with 0.05% trypsin/EDTA for 5 min, and the dissociation process was terminated using fresh medium composed of α-MEM with 10% FBS, 100 U/mL of penicillin, and 100 mg/mL of streptomycin. These dissociated cells were used for all subsequent experiments. 

### 4.4. Suspension Cell Culture and Conditioned Medium (CM) Collection

Dual 3D spheroid ASCs were cultured for 3 days in low-glucose DMEM containing 10% FBS, 100 U/mL of penicillin, and 100 mg/mL of streptomycin (Gibco) using a spinner flask (Thermo Fisher) and a magnetic stir plate. The CM of each sample was then subjected to centrifugation at 1000× *g* for 10 min, and the resulting supernatants were collected and used for the subsequent experimental study.

### 4.5. Quantitative Reverse Transcription (qRT)–Polymerase Chain Reaction (PCR) Analysis

A qRT–PCR assay was conducted using the method outlined by Kim et al. [16]. Briefly, total RNA was extracted from each cell using RNA-stat (Iso-Tex Diagnostics, Friendswood, TX, USA). The isolated RNA was subsequently reverse-transcribed using Taqman Reverse Transcription Reagents (Applied Biosystems, Foster City, CA, USA) according to the manufacturer’s instructions. The synthesized cDNAs were subjected to qRT-PCR using human-/mouse-specific primers and probes. RNA levels were examined using the ABI PRISM 7000 Sequence Detection System (Applied Biosystems, Foster City, CA, USA). The relative mRNA expression of each experimental group was calculated by normalizing the gene expression levels to the reference gene glyceraldehyde-3-phosphate dehydrogenase (GAPDH). The primers used for qRT-PCR are shown in Table 1. All primer/probe sets used for qRT-PCR were purchased from Applied Biosystems.

### 4.6. Matrigel Tube Formation Assay

To assess the tube formation potential, CMs were collected with modifications, as previously described [16]. For the tube formation assay, HUVECs at a concentration of 1 × 10^4^ cells/well were embedded in low-glucose DMEM (Gibco) containing 1% FBS (control) or the CM of SVFs and ASCs in basement membrane matrix gel (Matrigel, BD)-coated glass slides (NUNC). After incubation for 5 h, representative fields were randomly photographed using inverted microscopy, and the tube length and branching points were measured using the method outlined by Kim et al. [16].

### 4.7. Scratch Migration Assay

Scratch migration assays were performed following the method reported by Jeong et al. [39]. Briefly, HDFs were seeded at a density of 1 × 10^5^ cells per well in 24-well culture plates and incubated at 37 °C with 5% CO_2_ for 27 h to form confluent monolayers. The monolayers were then scratched using a sterile pipette tip to create wounds and treated with the CM of each cell. To assess cell mobility and the wound closure effect, images were captured at 5 random areas within the scratched areas. The NIH Image program (http://rsb.info.nih.gov/nih-image/ (accessed on 1 July 2022)) was used for the analysis of the scratch wound area.

### 4.8. Matrigel Plug Assay

To investigate the in vivo vasculogenic potential, we performed a Matrigel plug assay following the procedure outlined by Jeong et al. [39]. Specifically, 2 × 10^5^ cells were mixed with 500 μL of Matrigel and subcutaneously transplanted into nude mice. After 14 days, the Matrigel plugs were collected and hemoglobin levels were evaluated using Drabkin’s Reagent Kit (Sigma, St. Louis, MO, USA).

### 4.9. Cell Transplantation in the HLI Mouse Model

The experimental protocols used in this study were approved by the Catholic Kwandong University Institutional Animal Care and Use Committee. HLI was induced using the method described by Kim et al. [16]. Briefly, male nude mice aged between 7 and 10 weeks, weighing 18–22 g, were used for the experiments (Joongang Laboratory Animal Inc., Seoul, Republic of Korea). Each mouse was anesthetized with isoflurane (induction: 450 mL of air, 4.5% isoflurane; maintenance: 200 mL of air, 2.0% isoflurane; Baxter International, Inc., Deerfield, IL, USA), and the right femoral artery was surgically ligated. Subsequently, a solution containing 1 × 10^6^ cells was intramuscularly injected into the ischemic hind limb area after surgery (n = 7 for each group). Euthanasia was carried out by intravenous injection of thiopental-sodium (40 mg/kg). To assess the blood flow in the hind limb over time, laser Doppler perfusion imaging (Moor Instruments, Axminster, UK) was used.

### 4.10. Histological Analysis

For histological analysis, adductor muscles were harvested and fixed in paraformaldehyde for 4 h, followed by overnight incubation in a 15% sucrose solution. The tissues were then embedded in OCT compound (Sakura Finetek USA, Torrance, CA, USA) and cut into 10-μm-thick sections [16]. Frozen sections of ischemic tissues were stained with biotinylated isolectin B4 (ILB4, 1:250; Vector Laboratory Inc., Burlingame, CA, USA) primary antibodies. This was followed by incubation with streptavidin Alexa Fluor 488 (1:400; Invitrogen, Waltham, MA, USA) secondary antibodies for capillary density measurement. Subsequently, five fields from five tissue sections were randomly selected, and the number of capillaries was counted.

### 4.11. Statistical Analysis

Student’s *t*-test was used to make comparisons between two groups. For comparisons involving more than two groups, we used one-way analysis of variance (ANOVA) followed by Bonferroni’s multiple comparison test. Statistical significance was set at *p* < 0.05.

## Figures and Tables

**Table 1:** Primers for qRT-PCR.

Gene	Human	Mouse
VEGF-A	Hs99999070_m1	Mm01204733_m1
FGF-2	Hs00266645_m1	Mm00433287_m1
HGF	Hs00300159_m1	Mm01135184_m1
IGF-1	Hs01547657-m1	Mm00439560_m1
GAPDH	Hs99999905_m1	Mm99999915_g1
SDF-1	Hs00171022_m1	
GCP-2	Hs00237017_m1	

## Data Availability

The data presented in this study are available on request from the corresponding author upon reasonable request.
